# *Athyrium multidentatum* (Doll.) Ching extract induce apoptosis via mitochondrial dysfunction and oxidative stress in HepG2 cells

**DOI:** 10.1038/s41598-017-02573-8

**Published:** 2017-05-23

**Authors:** Guoyuan Qi, Zhigang Liu, Rong Fan, Ziru Yin, Yashi Mi, Bo Ren, Xuebo Liu

**Affiliations:** 1 Laboratory of Functional Chemistry and Nutrition of Food, College of Food Science and Engineering, Northwest A&F University, Yangling, Shaanxi 712100 China; 2 Department of Nutrition and Health Sciences, University of Nebraska-Lincoln, Lincoln, NE 68503 USA; 3 Department of Bioscience and Bioengineering, Hebei University of Science and Technology, Shijiazhuang, 050000 Hebei China

## Abstract

*Athyrium multidentatum* (Doll.) Ching (AMC), a unique and nutritious potherb widely distributed in china, has been extensively used in traditional Chinese medicine. Previous studies indicated that AMC extract exhibited antioxidant and antitumor properties. However, the chemical composition of AMC and molecular mechanism of AMC toxicity to HepG2 cells have not yet been elucidated. Hence, this study aimed to investigate the chemical compositions and the underlying mechanisms of the antiproliferative and apoptotic effects of AMC on HepG2. HPLC-MS analysis showed that AMC contain five compounds with chlorogenic acid accounting for 43 percent. Also, AMC strongly inhibited the cell growth and induced apoptosis and cell cycle arrest in HepG2 cells by significantly upregulating the protein expressions of Fas, Fas-L, Bax/Bcl-2, cyto-*c*, cleaved caspase-3, and PARP in a dose-dependent manner, which indicates AMC induces apoptosis in HepG2 cells through both intrinsic and extrinsic pathways. Moreover, AMC provoked the production of ROS, H_2_O_2_, and NO, modulating the PI3K/Akt, MAPK, NFκB and Nrf2 pathways and their downstream transcriptional cascades, ultimately evoked oxidative stress and apoptosis in HpeG2 cells. Further *in vivo* experiments demonstrated that AMC significantly suppressed the tumor growth, suggesting that AMC may be a novel promising agent for hepatocellular carcinoma treatment.

## Introduction

Hepatocellular carcinoma (HCC), the predominant primary liver cancer, is the fifth most frequent cancer worldwide and the second most common cause of cancer death in the world^1, 2^. However, Chemotherapy, as one of the cancer therapeutic methods, possesses side effects despite the significant advances in treatment of hepatocellular carcinoma^3^. Therefore, the development of an effective cancer chemotherapeutic agent with fewer side effects has been an urgent need for treatment of hepatocellular carcinoma. Apoptosis is a programmed cell death, which is critical in both normal development and maintenance of body homeostasis. Therefore, the induction of apoptosis will be a possible effective approach to alleviate hepatocellular carcinoma^4^.

It has been identified that apoptosis is associated with two major routes, including the cell death receptor-mediated extrinsic pathway and the mitochondria-mediated intrinsic pathway^5^. Particularly, the mitochondrial pathway mainly participates in phytochemicals-induced cancer cells apoptosis^6^. The mitochondrial-mediated apoptotic pathway begins with mitochondrial membrane potential loss, cytochrome c release, the executioner caspase-3 cleavage, and eventually resulting in the formation of apoptotic bodies. Also, the activation of mitogen protein kinases (MAPK) is involved in apoptosis processes. There are three major MAPK pathways in the extracellular signal-regulated kinases: ERK1/2 (p44/p42), c-Jun amino-terminal kinase JNK (p46/p54) and p38 kinase. In addition, the PI3K/Akt signaling pathway also plays a crucial role in carcinogenesis and tumor progression by inhibition of apoptosis and promoting cell proliferation^7^.

Natural phytochemicals are considered as good sources of potential cancer chemopreventive and chemotherapeutic agents. Pharmaceuticals derived from plants have played an important role in the health care in both ancient and modern times. Recently, great attention has been paid to the highly effective phytochemical antioxidants and agents from natural sources^8^.

*Athyrium multidentatum* (Doll.) Ching (AMC) is a common fern species in northeast China, especially the Changbai Mountain area. The potential utilization of AMC as medicine has been documented in traditional Chinese medicine as a tranquilizer, antihypertensive, and diuretic^9–11^. Liu *et al*. reported that all fractions of polysaccharides extracted from AMC possessed considerable antioxidant activity^12^. Sheng *et al*. assessed antioxidant activity of degraded polysaccharides from AMC and found all four low molecular polysaccharides exhibited antioxidant activity *in vitro* systems^13^. Our previous research demonstrated that AMC was a quality nutrition source for proteins, carbohydrates, fat, and minerals. AMC extracts possessed a strong antioxidant activity, protective effects on biomolecules, cellular antioxidant activity (CAA), and anti-proliferative effects owing to its highest total phenolic (476.52 ± 11.26 mg GAE per gram extract) and total flavonoid (924.81 ± 4.25 mg RNE per gram extract) contents. Furthermore, AMC extracts exhibited a promising effect on the inhibition of cell proliferation and stimulated apoptosis in HepG2 cancer cells^10^. However, the underlying molecular mechanisms of AMC-induced apoptosis in HepG2 cells remain elusive.

In this study, we aimed to investigate the anticancer effects of AMC on human hepatocellular carcinoma HepG2 cells and the underlying molecular mechanisms. AMC has been proved to induce HepG2 cell apoptosis via the death receptor-mediated extrinsic pathway and mitochondria-mediated intrinsic pathway. AMC triggered cancer cell death via apoptosis-related PI3K/Akt, MAPK, and p53 pathways. Furthermore, the nuclear translocation of NFκB and Nrf2 oxidative stress-dependent pathways were also involved in AMC-induced apoptosis in HepG2 cells. Additionally, AMC administration induced G2/M phase cell cycle arrest by manifesting decreased cell-cycle related protein expressions of CDK1, CDK2, and Cyclin D1. AMC also displayed significant inhibitory effects on tumor size *in vivo*. Taken together, our results indicated that AMC triggers apoptosis via mitochondrial dysfunction and oxidative stress in HepG2 cells, significantly impeding tumor growth, and may provide a new strategy for dietary interventions of hepatocellular carcinoma patients.

## Results

### Identification and quantification of compounds of AMC

**Figure 1:**
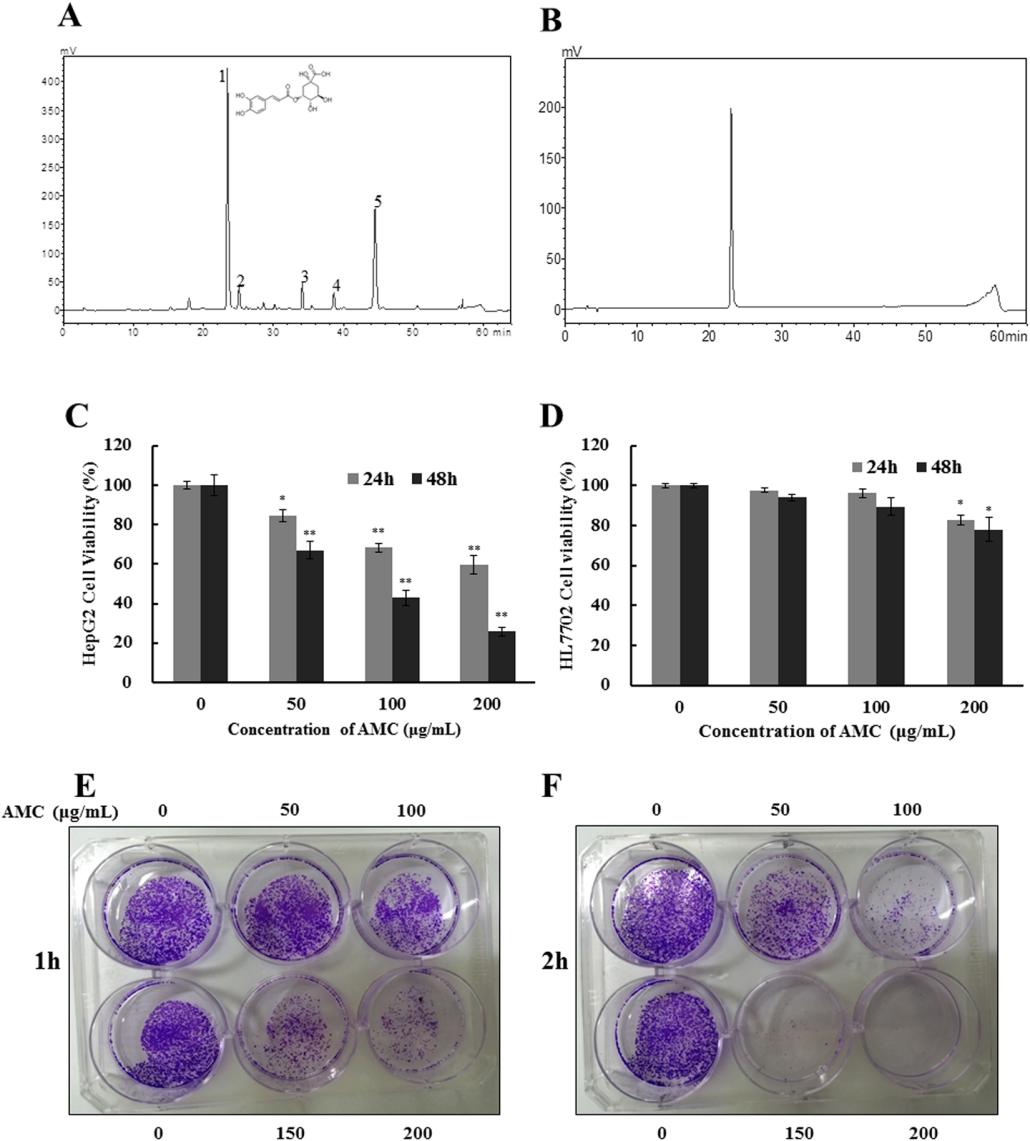
Composition analysis and effect of AMC on cells viability and clonogenicity. HPLC chromatogram (obtained at 280 nm) of *Athyrium multidentatum* (Doll.) Ching extract (AMC) (1509 μg/mL) (**A**) and standard Chlorogenic acid (160 μg/mL) (**B**). HepG2 (**C**) and HL7702 (**D**) cell viability was assessed by MTT assay after treated with various concentrations of AMC for 24 h or 48 h. HepG2 cells were seeded in six-well plates. After 24 h of incubation, cells were treated with various concentrations of AMC for 1 h (**E**) or 2 h (**F**) and subsequently allowed to grow into colonies for 10 days. The results are presented as mean ± SD, n ≥ 6 wells per group, *p < 0.05 and **p < 0.01 versus the control group.

HPLC chromatograms exhibited that the five major peaks of the AMC had retention time of 22.976 min (Peak 1), 24.652 min (Peak 2), 33.758 min (Peak 3), 38.133 min (Peak 4), 44.019 min (Peak 5). The Peak1 had the same retention time with that of chlorogenic acid in HPLC experiments (RT = 22.934 min) (Fig. 1A and B), which accounted for 43 ± 0.99% of AMC. Next, HPLC-MS was performed to confirm and determine that chlorogenic acid presence and the molecular formula of other compounds in AMC. Table 1 displayed their retention times, formula, wavelengths of maximum absorption in the visible region, mass spectral data and molecular formula. Peak 1(RT = 22.934), with [M-H]^+^ at 355, MS^2^ fragment at m/z 163, was tentatively identified as chlorogenic acid. Peak 2 presented the same pseudomolecular ion [M-H]^+^ at m/z 355 and MS^2^ fragmentation patterns with peak1 was tentatively identified as an isomer of chlorogenic acid. Peak 3 presented a pseudomolecular ion [M-H]^+^ at m/z 451, releasing an MS^2^ fragment at m/z 289, loss of a hexosyl moiety which was consistent with eriodictyol O-hexoside, although the nature and position of the sugar residue could not be established. From the ESI^+^ ion mode we found that decarboxylation reaction happened in Peak 5, suggesting that this compounds contained carboxyl. Therefore, peak 5 was temporarily identified as a caffeic acid amide, N-caffeoyl-phenylalanine. Nevertheless, peak 4 (RT = 38.133 min, λ_max_ = 282 nm, [M-H]^+^ at m/z 435) remained unidentified.

**Table1:** Retention times (RT), wavelengths of maximum absorption in the visible region (λ_max_), mass spectral data and molecular formula of the substances in AMC.

Compound	RT (minutes)	λ_max_ (nm)	MS^+^ (m/z)	MS^2^ fragment ions (m/z)	Molecular Formula
1	22.976	216, 325	355	163	C_16_H_18_O_9_
2	24.652	325	355	163	C_16_H_18_O_9_
3	33.758	283	451	289	C_21_H_22_O_11_
4	38.133	214, 282	435	273	C_21_H_22_O_10_
5	44.019	217, 277	328	103, 131, 152, 282	C_18_H_17_NO_5_

### Effect of AMC on HepG2 cells viability

The MTT assay was performed to evaluate the effect of AMC on cell proliferation of HepG2 cancer cells. As shown in Fig. 1C, treatments of 50, 100, and 200 μg/mL AMC for 24 h decreased the HepG2 cells viability to 84.5%, 68.3%, and 59.6%, respectively. After being incubated for 48 h, the cell viability was decreased to 67.09%, 42.92%, and 25.72%, respectively. The IC50 values of AMC on HepG2 cells were 220.32 ± 10.32 μg/mL after 24 h and 113.51 ± 7.45 μg/mL after 48 h. To investigate the cytotoxicity in normal cells, the same concentration of AMC treatment was performed on human liver cells HL7702 for 24 h and 48 h, respectively (Fig. 1D). AMC had no significant toxic effects on the viability of HL7702 cells, which illustrated the experimental conditions in this study had no toxicity to normal human liver cells. The IC50 values of AMC on HL7702 cells were 332.25 ± 15.17 μg/mL after 24 h and 303.98 ± 20.68 μg/mL after 48 h. To further determine the impacts of AMC on cancer cell growth, the colony formation assay was conducted on HepG2 cells (Fig. 1E and F). AMC showed potent and dose-dependent inhibition effects on the colony formation of HepG2 cells even after treated for 1 h or 2 h.

### AMC elicited extrinsic and intrinsic apoptosis in HepG2 Cells

**Figure 2:**
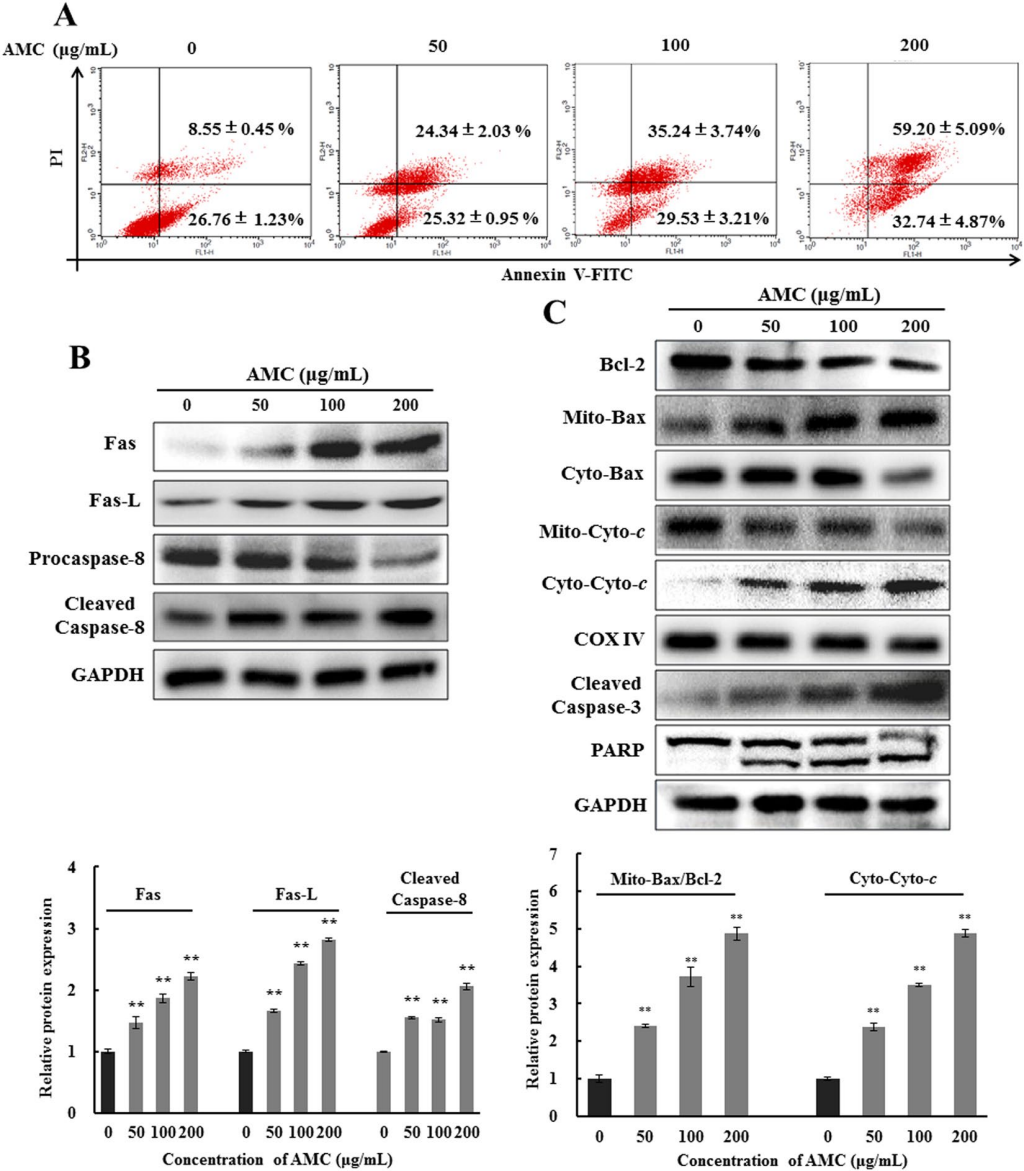
AMC elicited extrinsic and intrinsic apoptosis in HepG2 Cells. Cells were incubated with AMC at indicated concentration for 24 h before tests were performed. Apoptotic cells detected by flow cytometry with annexin V conjugated with PI staining. (**A**) The Fas, Fas-L, procaspase-8 (**B**), Bcl-2, Bax, Cyto-*c*, Cleaved Caspase-3 and PARP. (**C**) expressions of the HepG2 cells were analyzed by western blot. The data were shown as the mean ± SD (n = 3). *p < 0.05 and **p < 0.01 versus the control group.

To confirm the effects of AMC treatment on apoptosis, HepG2 cells were stained with Annexin V-FITC/PI, and subsequently analyzed by flow cytometer. Results implied that the early apoptosis rate increased in a time-dependent manner after AMC treatment (Fig. 2A), suggesting that AMC can induce apoptosis in HepG2 cells.

The extrinsic pathway of apoptosis is initiated by death ligands, such as Fas-ligand (Fas-L), leading to cleavage of procaspase-8 to its active form. As shown in Fig. 2B, the expression of Fas and its ligand Fas-L was observed upon after treatment with various concentrations of AMC for 24 h. In addition, it was also found that AMC significantly decreased the expression level of procaspase-8 and increased cleaved caspase-8 protein expression in the cytosol.

Mitochondria play a pivotal role in the intrinsic pathway of apoptosis^14^. Previous studies demonstrated that AMC could substantially suppress mitochondria membrane potential (MMP)^10^. As the early stage of intrinsic apoptosis, loss of MMP could induce cytochrome *c* releasing into cytosol from mitochondria membrane and activating caspase cascade and PARP cleavage. We investigated whether AMC can induce cytochrome *c* release from mitochondria by influencing the translocation of Bax, a key protein in the induction of cell apoptosis. As shown in Fig. 2C, an increase of cytochrome c in the cytosol and a decrease of mitochondrial cytochrome *c* levels were detected after 24 h of exposure to various concentrations of AMC, compared with the control group. AMC also suppressed the expression level of apoptotic protein (Bcl-2) and triggered the translocation of Bax from cytoplasm to mitochondria, and the Bcl-2/Mito-Bax ratio gradually decreased in a concentration-dependent manner. We further confirmed whether Bax translocation from the cytoplasm to the mitochondria and cytochrome *c* release from mitochondria induced the caspase pathway. As shown in Fig. 2C, the expression levels of cleaved caspase-3 and the cleavage of PARP were increased in a dose-dependent manner after treatment with AMC for 24 h. These results suggested that AMC-induced apoptosis in HepG2 cells was activated through the death receptor-mediated extrinsic pathway and mitochondria-mediated intrinsic apoptotic pathway.

### AMC induced oxidative stress in HepG2 cells

**Figure 3:**
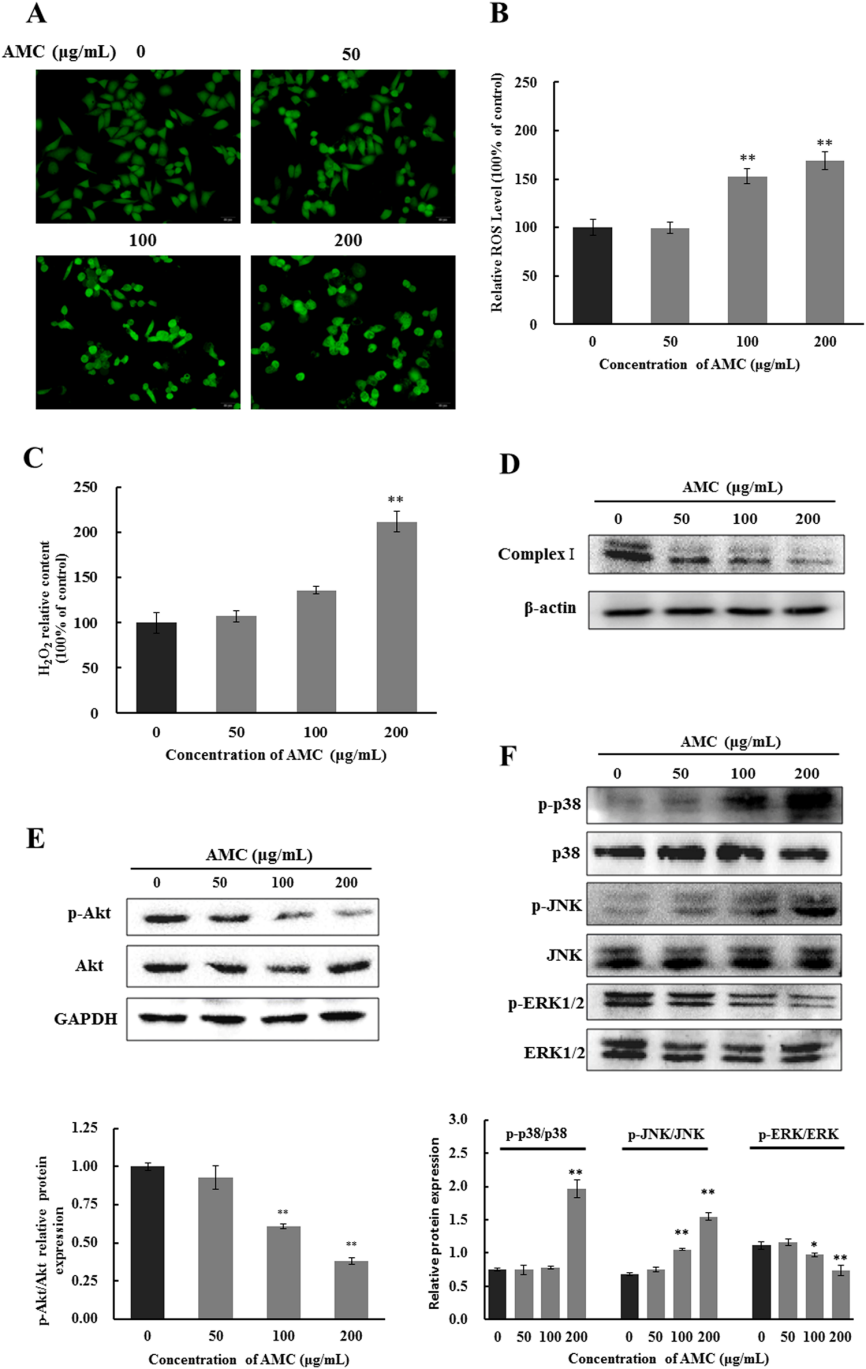
Effect of oxidative stress, PI3K/Akt, and MAPK pathways on AMC induced apoptosis in HepG2 cells. Cells were exposed to AMC (50, 100 and 200 μg/mL) for 24 h followed by incubation with DCFH-DA at 37 °C for 30 min and then detected by a fluorescence microscope (**A**). The ratio of fluorescence intensity (**B**) was obtained using Multimode plate readers. H_2_O_2_ was measured, after cells were incubated with variable concentrations of AMC for 24 h (**C**). Protein expression of Complex-I (**D**), Akt, p-Akt (**E**) p-p38, p38, p-JNK, JNK, p-ERK1/2, and ERK1/2 (**F**) were analyzed by western blot. Values are means ± SD for at least three independent experiments performed in triplicate. *p < 0.05 and **p < 0.01 versus the control group.

The cytotoxic effect of AMC was hypothesized to be related with oxidative stress. To confirm and quantify the oxidative stress triggered by AMC in HepG2 cells, the production of ROS and H_2_O_2_ was measured by DCFH-DA method and Hydrogen peroxide assay kit. As shown in Fig. 3, AMC significantly increased the production of ROS and H_2_O_2_ of HepG2 cells in a dose-dependent manner. In addition, AMC dose-dependently reduced the expression of Complex I (Fig. 3D).

### AMC induced apoptosis via mediating PI3K/Akt and MAPK pathways

PI3K/Akt is a potential survival signaling pathway involved in the regulation of the cell cycle, growth, survival, metabolism, and tumorigenesis^15, 16^. To evaluate the effect of AMC on PI3K/Akt pathway in HepG2 cells, the p-Akt and Akt were detected by western blot assays. When treated with 100 or 200 μg/mL of AMC, the amount of p-Akt significantly decreased, compared with control (p < 0.01). However, the expression of total Akt was not affected (Fig. 3E). In comparison to the control group, PI3K/Akt inhibitor LY294002 significantly increased cleaved caspase-3 and PARP cleavage in both the presence and absence of AMC (Supplementary Fig. E).

Accumulating evidence indicated that MAPK signaling pathway, the most extensively investigated molecular pathway in human cancer, is associated with apoptosis. In order to investigate whether MAPK signal pathway was involved in AMC induced cell apoptosis, the protein expressions of phosphorylated MAPK (ERK1/2, JNK and p38) were detected. From Fig. 3F, AMC significantly restrained the phosphorylation of ERK1/2, and increased the expressions of p-JNK and p-p38 in a dose-dependent manner, while the total ERK1/2, JNK and p38 protein levels remained constant throughout the process of AMC treatment. As shown in Supplementary Fig. B and C, both the JNK inhibitor SP600125 and the p38 inhibitor SB203580 increased cleaved caspase-3 levels and PARP cleavage levels respectively and in the presence of AMC. In addition, the ERK inhibitor U0126 markedly increased cleaved caspase-3 levels and PARP cleavage in the absence of ISO and promoted the activation effects of AMC on those protein levels (Supplementary Fig. D). Taken together, these results indicated that AMC induced apoptosis in HepG2 cells through PI3K/Akt and MAPK pathways.

### Effect of NFκB and p53 signaling pathways on AMC induced apoptosis

**Figure 4:**
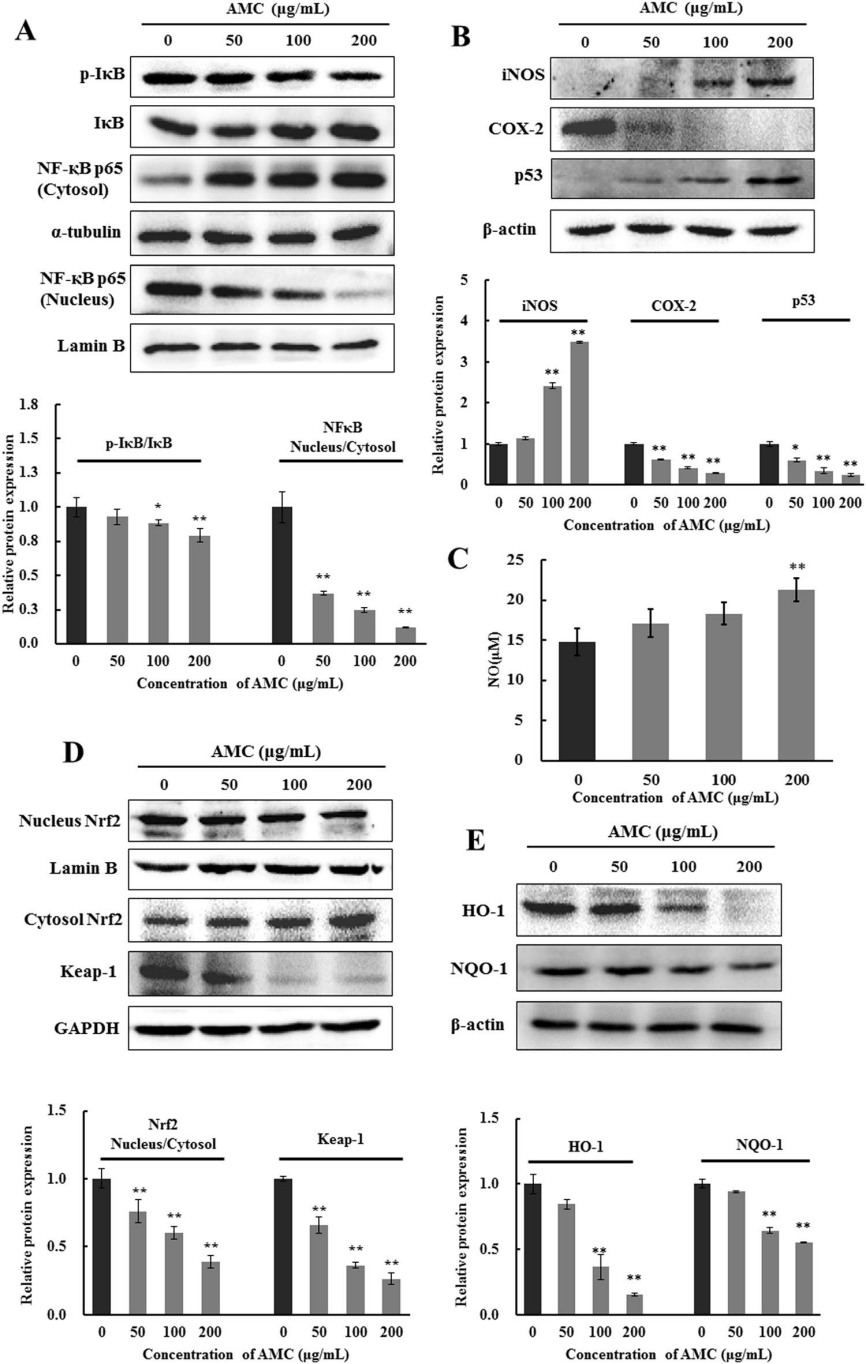
AMC induced apoptosis via NFκB, p53, and Nrf2 related signaling pathways in in HepG2 cells. Cells were incubated with AMC at indicated concentration for 24 h before tests were performed. Protein expression of the NFκB signaling pathway (**A**), iNOS, COX-2, p53 (**B**), Nrf2 signaling pathway (**D**), HO-1, and NQO-1 (**E**) were analyzed by western blot. GAPDH and lamin B were used as internal controls. Nitrite (a stable end product of NO) present in culture medium was detected by the Griess reaction (**C**). Values were presented as mean ± SD for at least three independent experiments performed in triplicate. *p < 0.05 and **p < 0.01 versus the control group.

Nuclear factor-κB (NFκB) is a transcription factor involved in cell survival, adhesion, inflammation, differentiation, and growth^17^. This study also discussed whether NFκB signaling pathway was involved in AMC-induced apoptosis of human hepatocellular carcinoma HepG2 cells. As shown in Fig. 4A, after treated with various concentrations (0, 50, 100, 200 μg/mL) of AMC for 24 h, the AMC groups cells displayed a significant decrease protein expression levels of p-IκB and NFκB in nucleus, while the protein expression of NFκB in cytosol remarkably increased. Additionally, the NFκB inhibitor pyrrolidinedithiocarbamic acid (PDTC) significantly increased cleaved caspase-3 expression and PARP cleavage in the absence of AMC and promoted the activation effects of AMC on those protein levels (Supplementary Fig. F). These results confirmed that NFκB signaling pathway was involved in AMC-induced apoptosis of HepG2 cells. Furthermore, expressions of iNOS, p53, and NO were markedly up-regulated, whereas COX-2 was decreased after AMC treatment for 24 h in HepG2 cells (Fig. 4B and C).

### Effects of AMC on the Keap1/Nrf2 transcriptional pathway

To investigate whether Nrf2-dependent antioxidant defense mechanism was activated during AMC-induced oxidative stress mediated cytotoxicity in HepG2 cells, we examined the Nrf2 nuclear translocation and the protein expressions of HO-1 and NQO-1. As shown in Fig. 4D, compared with the control group, AMC significantly inhibited the nuclear translocation of Nrf2 and expression of Keap1 in HepG2 cells. Furthermore, the results showed that AMC decreased the protein expression of HO-1 and NQO-1 in a dose-dependent manner in HepG2 cells (Fig. 4E).

### Effect of AMC on cell cycle controlling of HepG2 cells

**Figure 5:**
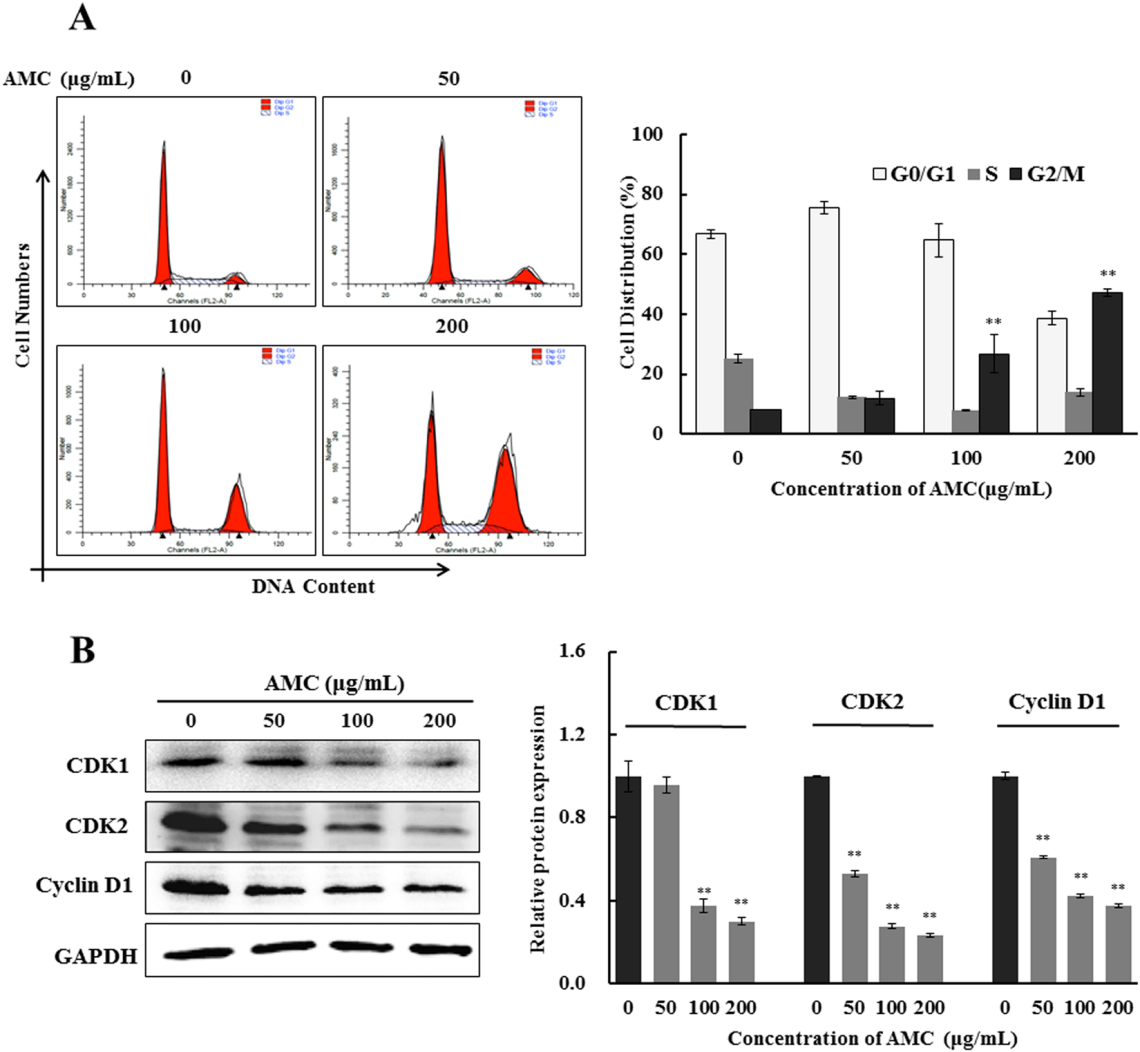
Effect of AMC on cell cycle arrest of HepG2 cells. Representative flow cytomeric analysis for the DNA content in HepG2 cells treated with AMC at different concentrations of 50 μg/mL, 100 μg/mL, and 200 μg/mL. Cell cycle distribution and cell number percentage in each phase (G0/G1, S, and G2/M) were detected and calculated (**A**). The protein expression of CDK1, CDK2, and Cyclin D1 were analyzed by western blot (**B**). Values were presented as mean ± SD for at least three independent experiments performed in triplicate. *p < 0.05 and **p < 0.01 versus the control group.

Apoptosis and cell cycle arrest are two main mechanisms for cell growth inhibition. The cell cycle distribution was investigated by flow cytometry flow on HepG2 cells with or without treatment with AMC for 24 h. As shown in Fig. 5A, the AMC treatment resulted in an increase in the percentage of cells in the G2/M phase in a concentration-dependent manner. In light of the above finding of G2/M arrest of cells after AMC exposure, expression levels of a series of regulatory proteins related to cell cycle were examined in HepG2 cells. Protein expressions of CDK1, CDK2 and Cyclin D1 were both markedly decreased (Fig. 5B). Overall, these data suggested that AMC could induce cell cycle arrest in G2/M phase associated with decreases of CDK1, CDK2 and Cyclin D1.

### AMC attenuated tumor growth *in vivo*

**Figure 6:**
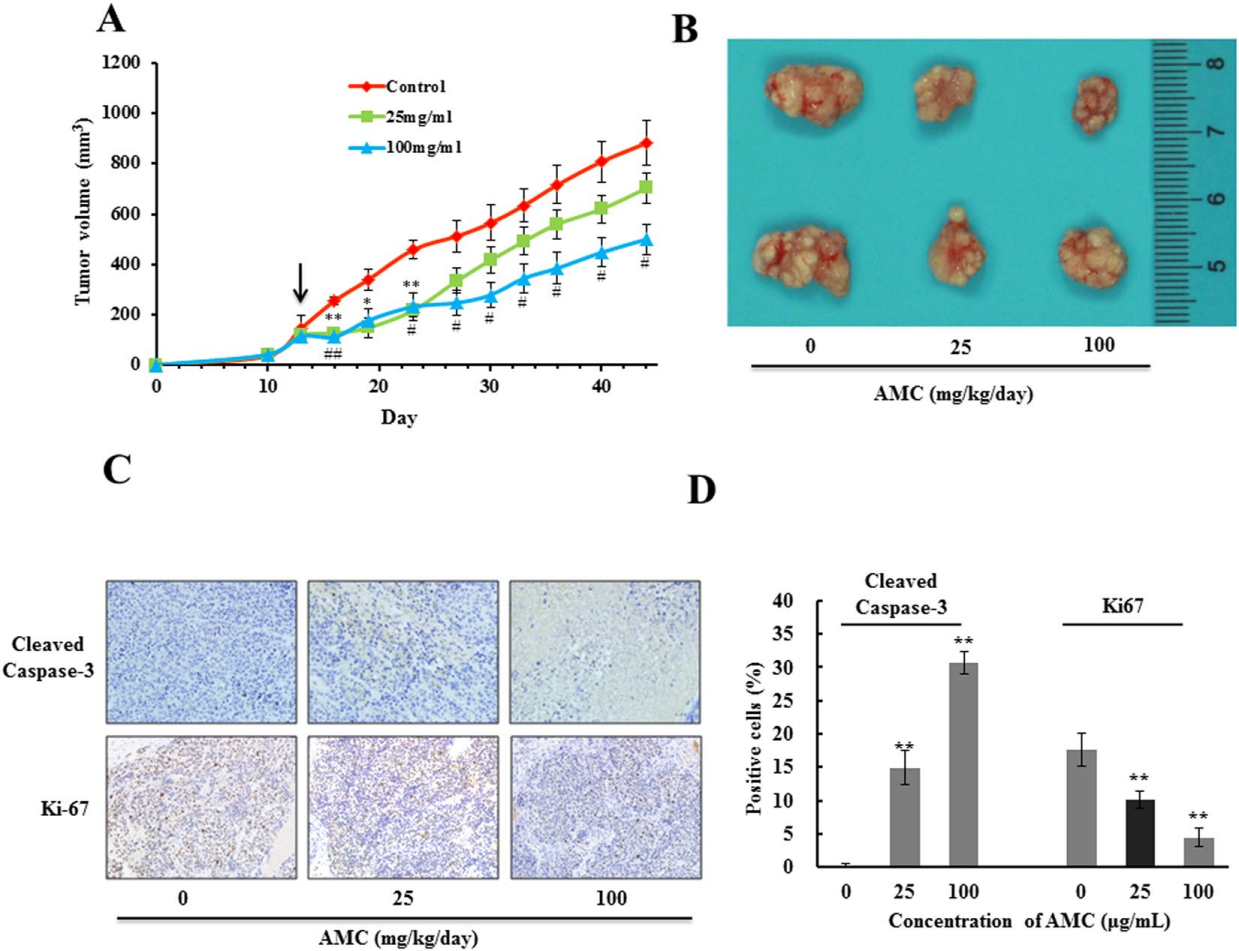
Growth inhibition of transplantable tumors by AMC *in vivo*. The average tumor volume calculated at the certain time points in HepG2-bearing Balb/c nu/nu mice (n = 5, per group) (**A**). *p < 0.05 and **p < 0.01 25 mg/kg AMC-treatment group versus the control group. ^#^p < 0.05 and ^##^p < 0.01 100 mg/kg AMC-treatment group versus the control group. Tumors were isolated on day 44, tumors formed in each group were photographed and 2 representative tumors from 5 different mice are displayed (**B**). Values are presented as means ± SEM for at least three independent experiments performed in triplicate. Immuno staining was performed on paraffin embedded tissue sections for cleaved caspase-3 and Ki-67 using respective antibodies in control tumor tissues and AMC treated tumor tissues after 30th day of treatment. Final magnification shown is 200× (**C**). (**D**) The images of IHC were quantified by Image J and values were presented as mean ± SD for at least three independent experiments performed in triplicate. *p < 0.05 and **p < 0.01 versus the control group.

To further evaluate whether AMC has an effect to inhibit tumor growth *in vivo*, we measured the tumor volume in a xenograft tumor model in which HepG2 cells were injected subcutaneously into nude mice. AMC (25 or 100 mg/kg) were selected for *in vivo* experiments. When transplant tumors reached a mean group size of approximately 100 mm^3^ (the thirteenth day), mice were treated with AMC every day for 31 days. Results clearly indicated that compared with the control group, AMC showed a significant inhibitory effect on tumor size (Fig. 6A and B). Caspase-3 and Ki-67 staining confirmed an increased apoptosis and a reduction in proliferative cells in AMC treated tumor animals in comparison to the untreated control (Fig. 6C).

## Disscussion

Hepatocellular carcinoma (HCC) is a chronic disease affecting millions of humans^18^. However, the conventional therapies are not effective enough to treat patients of HCC. Therefore, it is imperative to find novel and effective strategies with low toxicity for the treatment of HCC. In recent years, Traditional medicinal plants possessing chemotherapeutic and chemopreventive properties have been investigated for their possible clinical application in the prevention and treatment of cancer and minimal side effects^19–21^. Extracts isolated from Korean *Lonicera japonica* Thunb., mulberry, *Tetrastigma hemsleyanum*, and *Costus speciosus* all displayed a promising pro-apoptosis effect on HepG2 cancer cells^22–24^.

AMC is a unique and nutritious potherb in Changbai Mountain area and widely used as an effective folk medicine for conditions such as high blood pressure, anxiety and arthritis^12, 13^. Our previous studies demonstrated that AMC exhibited excellent antioxidant activity, anti-proliferative, and pro-apoptotic properties on HepG2 cells. Nevertheless, the underlying molecular mechanisms remain elusive. In this study, we further evaluated the anticancer effect of AMC in HepG2 cells. HPLC and HPLC-MS were performed to identify and quantify of compounds of the AMC. Five major components were detected in AMC, among which chlorogenic acid (Peak 1) accounted for 43% (Fig. 1A and B). We noticed that AMC significantly inhibited cellular growth of HepG2 in a dose-dependent manner after 24 h or 48 h treatment but revealed no obvious toxicity to HL7702 cells identified by MTT assay (Fig. 2C and D). The mechanism of differential toxic effects on cancer and normal cells is still need to be further explored. To further elucidate the role of AMC *in vivo*, a HepG2 tumor model in Balb/c nu/nu mice was established. Significant tumor suppression by AMC at concentration of 100 mg/kg was observed (Fig. 6A and B).

Apoptosis, a programmed cell death mediated through the death receptor-mediated extrinsic pathway and the mitochondrial-mediated intrinsic pathway, has been evaluated as a major mechanism to eliminate cancer cells^25^. The extrinsic apoptotic pathway is triggered by binding of Fas with its extracellular ligand, Fas-L^26^. Consequently, the Fas/Fas-L protein complex activates its procaspase-8, which proceeds to trigger procaspase-3, the vital enzyme for execution of the apoptotic process^27^. Our results revealed that AMC significantly promoted Fas and Fas-L expression level and inhibited procaspse-8 expression level in a concentration-dependent manner (Fig. 2B). These results indicated that HepG2 cells treated with AMC were subjected to apoptosis involving death receptor-mediated extrinsic pathway.

Activation of mitochondria-mediated intrinsic apoptotic pathway is also a key mechanism involved in the function of anti-tumor drugs. Mitochondria play a critical role in the regulation of various apoptotic processes, including drug-induced apoptosis^28^. Bcl-2 family members, such as pro-apoptotic protein Bax and anti-apoptotic protein Bcl-2 are critical regulators of the mitochondrial pathway^29, 30^. They can modulate the mitochondrial outer membrane permeability to control the release of mitochondrial cytochrome c^31^. In line with those findings, our study found that AMC significantly increased the translocation of Bax from cytoplasm to mitochondria and cytochrome *c* release from mitochondria in HepG2 cells (Fig. 2C). These processes led to the activation of the executioner caspases-3, and ultimately the inactivation of PARP. These data indicated that the mitochondrial pathway was involved in AMC-induced apoptosis.

ROS is an important intracellular signal of cell proliferation. Once intracellular ROS levels are too high, peroxidation damage and apoptosis will occur^32^. Various plant polyphenols exert their anticancer effects through inducing the generation of ROS^25, 32^. H_2_O_2_, a second messenger generated from mitochondria, regulates multiple cell signaling pathways and a range of cell functions such as inflammatory, apoptosis, and energy metabolism^33^. H_2_O_2_ acts as an efficient redox molecule since it can easily pass through the mitochondrial membranes and diffuse to other mitochondria^34^. AMC induced mitochondrial dysfunction and improved intracellular ROS and H_2_O_2_ production, thus increased oxidative stress in HepG2 cells (Fig. 3A–C). Complex I was generally considered a major site of ROS production where electron leaked and single electrons reacted with oxygen, producing superoxide anion^35^. Complex I dysfunction had often been linked with alteration in ROS levels^36^. AMC treatment remarkably decreased the expression of complex I in HepG2 cells (Fig. 3D). These results supported the notion that AMC can induce mitochondrial dysfunction in HepG2 cells.

Accumulated evidence suggested that PI3K/Akt and MAPKs signaling pathways were related to cellular redox-sensitive. PI3K/Akt pathway is a target for treating various tumors because it regulates cells proliferation and apoptosis^7^. Akt is the central downstream molecule of PI3K and its phosphorylation has been considered as an important factor in the prohibition of cancer. Akt is also involved in maintenance of the bioenergetic and metabolic capacities of mitochondria. Suppression of the phosphorylation of Akt by AMC was consistent with the loss of mitochondrial function (Fig. 3E). MAPK signaling pathway members played important roles in the induction of apoptosis. Once activated, ERK played an essential role in anti-apoptotic activity, while JNK and p38 were significant in pro-apoptotic activity^37^. Our results showed that AMC led to a decrease in phosphorylation of anti-apoptotic kinases ERK and an increase in phosphorylation of pro-apoptotic kinases JNK and p38, indicating that MAPKs signaling pathways were triggered to induce the apoptosis of HepG2 cells (Fig. 3F).

It is well established that NFκB is a crucial transcription factor associated with several cancer types and controls multiple genes involved in tumor progression such as cell proliferation and survival^38^. Mitochondria-derived H_2_O_2_ plays a crucial role in the activation of NFκB^39^. The tumor suppressor p53 is the key factor that maintains genomic stability by regulating the cell cycle and DNA repair process. P53 regulates the expression of inducible nitric oxide synthase (iNOS), which produces NO and promotes inflammation in tissues^34^. In present study, we found that AMC markedly increased p53 and iNOS levels and the protein expression of NFκB in the cytosol and decreased the level of NFκB in the nucleus which implied that AMC-induced apoptosis was associated with the NFκB pathway (Fig. 4A–C).

Nrf2 is a redox-sensitive transcription factor that plays a crucial role in modulating redox homeostasis and regulating inflammatory conditions through the induction of many stress responsive and cytoprotective enzymes and related proteins including HO-1 and NQO-1. Evidence accumulated during recent years suggests that HO-1 plays important roles in rapid tumor growth through anti-oxidative and antiapoptotic effects^40^. In this study, AMC treatment significantly decreased the expression of Nrf2 in nucleus, and subsequently downregulated HO-1 and NQO-1 levels in HepG2 cells (Fig. 4D and E). It has been reported that oxidants released from mitochondria can induce activation of Nrf2, leading Nrf2 to translocate into the nucleus and binding to the antioxidant response elements (ARE) of a range of phase II antioxidant defense genes^34, 41^. Moreover, activation of Nrf2 appears to be regulated by phosphorylation-signaling pathways, including MAPKs and PI3K^42^. In this study, we showed that AMC significantly suppressed ERK1/2 and Akt phosphorylation (Fig. 3E and F). These results suggested that Nrf2 pathway partly account for AMC-induced apoptosis in HepG2 cells.

Cell cycle arrest is a key mechanism by which anticancer drugs exert their effect on cancer cells. In the present study, after a 24 h exposure to AMC, cells showed a statistically significant increase in the G2/M fraction (Fig. 5A). This result aligned with findings by other authors who indicated the increases of the number of cells in G2/M of the cycle are related to apoptosis^43^. Cyclins and CDKs play important roles in the progression of tumor-associated cell cycle defects^44^. Cyclin D1 was proved to be associated with an increased transcription, translation and protein stability of CDKs in cancer cells^45^. Whereas CDK1 participated in cell cycle progression by enhancing cell cycle distribution in the G2/M fraction. The results of western blot analysis displayed that AMC caused sustained decreases in cell cycle related protein expressions of CDK1, CDK2 and Cyclin D1 in HepG2 cells (Fig. 5B). Numerous research studies have shown that p53 is associated with G2/M phase cell cycle arrest through the transcriptional activation of a CDK inhibitor, p21^44, 46–48^. p53 activation observed in this study may contribute to the reduction of CDK1 and CDK2, eventually leading to G2/M cell cycle arrest. Experimental results of xenograft tumor model and immunohistochemical (IHC) confirmed the data obtained in *vitro* to further emphasize the pro-apoptosis role of AMC in hepatocellular carcinoma (Fig. 6A–C).

**Figure 7:**
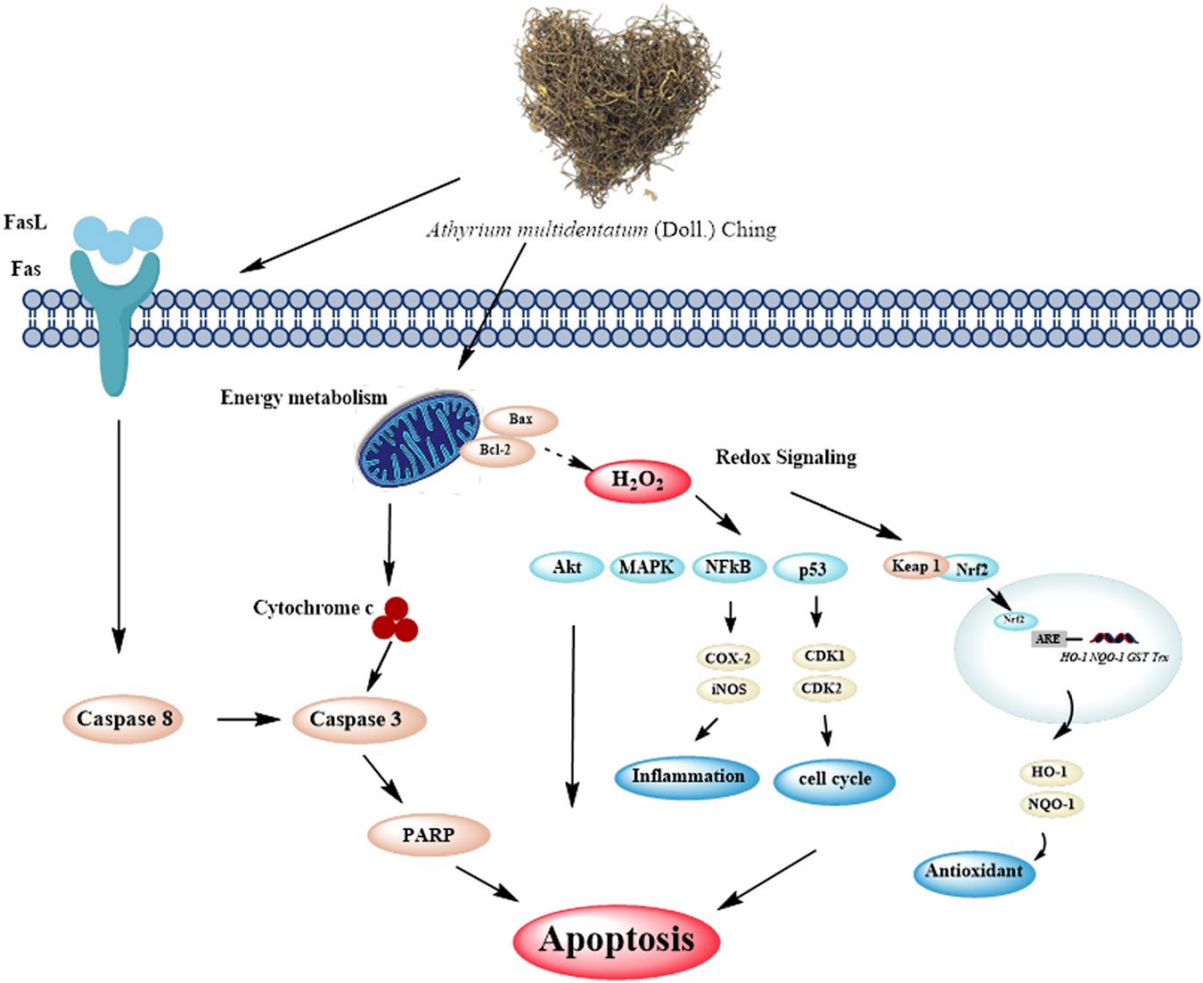
A schematic diagram of the proposed mechanisms of AMC-induced apoptosis in HepG2 cells.

In summary, our study was the first to identify the remarkable anticancer activity of AMC and offered a novel insight into the apoptotic mechanisms in hepatocellular carcinoma. It was demonstrated that AMC triggered apoptotic cell death in HepG2 cells through both intrinsic and extrinsic pathways via triggering mitochondrial-mediated H_2_O_2_ generation and regulating PI3K/Akt, MAPK, NFκB, Nrf2, and p53 pathways, ultimately led to mitochondrial dysfunction and oxidative stress. Furthermore, AMC showed a potential inhibitory effect on tumor size *in vivo*. A schematic diagram of these observed effects of AMC is shown in Fig. 7. The results of this study may provide a new option for the diet of susceptible hepatocellular carcinoma susceptible patients.

## Materials and Methods

### Cell culture and AMC treatment

HepG2 cells were provided by Kunming Institute of Zoology, Chinese Academy of Sciences (Kunming, China) and cultured in Roswell Park Memorial Institute-1640 supplemented with 10% FBS, 100 IU/mL penicillin, and 100 μg/mL streptomycin at 37 °C in a humidified atmosphere with 5% CO_2_. For the AMC treatment, AMC was dissolved in DMSO to a stock solution of 100 mg/mL and further diluted to different concentrations with FBS-free culture medium.

### Chemicals and reagents

For these experiments, 20, 70 - dichlorofluorescein diacetate (DCFH-DA), vitamin C, and linoleic acid (LA) were purchased from Sigma (Shanghai, China). Fetal bovine serum (FBS) and RPMI-1640 cell culture media were purchased from Thermo Fisher (Shanghai, China). The primary antibodies against Poly (ADP-ribose) polymerase (PARP), procaspase-8, cleaved caspase-3,NFκB, p65, CDK1, Cyclin D1, CDK2, IκB, p-IκB, Fas, Bcl-2, Bax, Cyto-*c*, and ND1 antibodies were provided by Cell Signaling Technology (Shanghai, China). α-tublin, lamin B, β-actin, GAPDH, HO-1, NQO-1, iNOS, COX-2, p53, Nrf2, keap-1 were from Santa Cruz Biotechnology (Dallas, Texas, USA). Cell cycle kit and Annexin V/PI cell apoptosis kit were from Wanleibio Company (Shenyang, Liaoning, China). All other chemicals were of analytical grade and were produced in China.

### Preparation of AMC

AMC was collected in Ji’an, Jilin province (China) from May 10th–16th, 2014. The bioactive components were extracted by the protocol from our previous report^10^.

### HPLC and HPLC-MS analysis

The phenolic and flavonoids-richen extract of AMC was resolved in 50% methanol (HPLC grade), filtered through a 0.45 μm pore-size membrane. Separation was performed using HPLC apparatus (LC-10Avp plus, Shimadzu, Japan) equipped with a reverse-phase C_18_ column (250 mm × 4.6 mm, 5 mm; Agilent, USA) and a UV detector at 280 nm. The mobile phase was solvent A (0.1% acetic acid in water) and solvent B (methanol) with a gradient elution: 0–5 min, B from 5–10%; 5–30 min, B from 10–40%; 30–50 min, B from 40–50%; 50–55 min, B from 50–98%; 55–56 min, B from 98–5% and maintained to 64 min. The flow rate was 1 mL/min, and the optical density of effluent was monitored at 280 nm.

For confirmatory purposes, high performance liquid chromatography (HPLC-MS) analysis was performed using HPLC system (Thermo scientific Ultimate 3000), coupled with a Mass Spectrometric Technology of Q Exactive (Thermo Fisher Scientific, Waltham, USA). AMC was separated by Thermo Scientific Hypersil GOLD C18 (100 × 2.1 mm, 1.9 μm) using flow rate at 0.3 mL/min at 35 °C. The mobile phase consisted of eluent A (0.05% formic acid) and eluent B (methanol) with a gradient elution: 0–2 min, 10% B; 2–8 min, B from 10% to 35%; 8–14 min, B from 35% to 95% and maintained to 18 min; 18–20 min, B from 90% to 10%. The injection volume was set at 10 μL. The MS were set as following conditions: positive ion mode, Atomization at 350 °C, sheath gas pressure 30 arb, aux gas pressure 10 arb, and scan range m/z 100 to 1500.

### Cell Viability Assay

HepG2 and HL7702 Cell viability was determined using the MTT assay. Cells were seeded in 96-well culture plates at a density of 2.5 × 10^4^ cells per well with 5% (*v*/*v*) CO_2_ at 37 °C. After 12 h of incubation, the growth medium was replaced by mediums containing different concentrations of the AMC (0–200 μg/mL) and incubated for 24 h or 48 h. Then, cells were incubated with MTT (0.5 mg/mL) for 4 h. Subsequently, 100 μL of DMSO was added to each well to dissolve the formazan crystals. Absorbance at 490 nm was measured with a microplate reader (Bio-Rad, China). Cell viability was presented as a percentage of the control group (untreated cells).

### Colony formation assay

Colony formation assays were performed as previously described with minor modifications. Cells were plated in 6-well plates at a concentration of 500 cells per well and were allowed to grow for overnight. Then, cells were incubated in the presence or absence of various concentrations (50, 100, 150, 200 μg/mL) of AMC for 1 h or 2 h. The media containing AMC was then removed, and the cells were washed with PBS and incubated for an additional 10 days in a complete medium to form colonies. Subsequently, cells were washed twice with PBS, treated with crystal violet for 10 min, washed three times with H_2_O, and then photographed with a digital camera.

### ROS, H_2_O_2_, and NO measurements

Cellular ROS was measured with DCFH-DA (dichlorodihydrofluorescein diacetate) which was employed to measure ROS production. Intracellular ROS in the presence of peroxidase can change DCFH to the highly fluorescent compound DCF. Hence, the detection of DCF fluorescence can represent the level of active oxygen produced by cells. After various treatments, DCFH-DA was then added to a final concentration of 10 μM and incubated for 30 min at 37 °C. Relative fluorescence intensities were monitored by a Multimode plate reader (Tecan, Switzerland) 485 nm excitation and 535 nm emission and were qualitatively analyzed by fluorescence microscopy (Olympus Optical, Japan).

H_2_O_2_ generation from cells were carried out using Hydrogen peroxide assay kit following the manufacturer’s instructions (Beyotime Institute of Biotechnology, Jiangsu, China).

NO production was determined by measuring the amount of nitrite (a stable end product of NO) present in culture medium based on the Griess reaction^47^. The culture supernatant was incubated with the same volume of the Griess reagent (0.1% (*w*/*v*) N-(1-naphathyl)-ethylenediamine and 1% (*w*/*v*) sulfanilamide in 5% (*v*/*v*) phosphoric acid) for 10 min at 37 °C. The absorbance was measured at 540 nm using the UV-2550 ultraviolet photometric scenery (Shimadzu, Japan). The amount of nitrite was calculated from a sodium nitrite standard curve.

### Flow cytometry assay

The distribution of cells in the different phases of the cell cycle was measured by the DNA content of nuclei labeled with propidium iodide (PI) according to the instructions of the manufacturer (Wanleibio, Shenyang, China). Briefly, 6 × 10^5^ cells were plated in 60-mm dishes and grown overnight, treated with various concentrations of AMC (0, 50, 100, 200 μg/mL) for 24 h. After added trypsin, cells were fixed in 70% ethanol overnight at 4 °C, incubated in 100 μL RNase A at 37 °C for 30 min, and stained with 500 μl propidium iodide at 4 °C for 30 min. Cell cycle distribution was monitored with a FACSCalibur (BD Biosciences, USA) and analyzed using CellQuest software.

Early and late phases of apoptotic cells were monitored by Annexin V-FITC/PI apoptosis detection kit. After treatment, cells were washed twice with cold PBS, re-suspended in binding buffer, and incubated with FITC and PI staining solution following the instructions of the manufacture (Wanleibio, Shenyang, China). Samples of 10,000 stained cells were analyzed by flow cytometry.

### Proteins extraction

The proteins in whole cell were extracted using RIPA lysis buffer. Nuclear and cytoplasmic proteins were extracted by using a nuclear and cytoplasmic protein extraction kit (Beyotime, Jiangsu, China). Briefly, after treatment, cells were washed twice with PBS, scraped and collected by centrifugation at 1500 × g for 5 min. Cell pellets were resuspended in 200 mL extraction buffer A and incubated for 15 min on ice. Afterwards, extraction buffer B was added, and samples were vortexed for 30 s at 4 °C. After centrifugation at 12000 × g for 5 min at 4 °C, supernatants, which contained the cytosolic fractions, were removed and stored at −80 °C until analyzed by gel electrophoresis. Pellets, which contained the nuclei, were resuspended in 50 mL of nuclear extraction buffer, and nuclear proteins were extracted by shaking the samples for 30 min at 4 °C. Afterwards, samples were centrifuged at 12 000 × g for 5 min at 4 °C. The supernatants were removed and analyzed using gel electrophoresis. The validation of the method used to isolate the cytosolic and nuclear fractions (Lamin B was used as a loading control for nuclear proteins, and α-tubulin was used as the loading control for cytoplasm proteins) was checked using western blot analysis (Supplementary Fig. G). Bax translocation from the cytoplasm to the mitochondria and cytochrome *c* release from mitochondria were evaluated by western blot analysis of cytosolic protein samples. Cytosolic and mitochondrial protein fractions were also prepared using the cell mitochondria isolation kit (Beyotime, Jiangsu, China) according to the manufacturer’s instructions.

### SDS-PAGE and western blot analysis

The SDS-PAGE analysis and western blot measurements were performed by previous reports from our lab^49, 50^.

### Animals and xenografts

Five-week-old male Balb/c nu/nu mice were purchased from Model Animal Research Center of Nanjing University (Nanjing, China). All of the experimental procedures followed by Guide for the Care and Use of Laboratory Animals: Eighth Edition, ISBN-10: 0-309-15396-4, and the animal protocol was approved by the animal ethics committee of Xi’an Jiaotong University. All surgery was performed under anesthesia and all efforts were made to minimize suffering. The mice were implanted subcutaneously in their right flanks with suspended in PBS. Tumors were established by injecting 5 × 10^6^ HepG2 cells into the right flank of Balb/c nu/nu mice. After tumor sizes reached approximately 100 mm^3^, mice were divided into two groups and intraperitoneal injection with saline or AMC at a dosage of 25 or 100 mg/kg for 30 days. The body weight of tumor-bearing mice was recorded every three days and tumor volume was calculated according to the formula ab^2^/2 (b is the smaller dimension of the two). Caspase-3 and Ki-67 staining was performed on AMC treated tissues sections as described before^48^.

### Statistical analysis

The data was representative of three independent experiments. The results were presented as the mean ± standard deviation (SD). Significant differences between measurements for the control and treated samples were analyzed using one-way factorial analysis of variance (ANOVA), followed by Tukey’s test (SPSS 16.0). A p < 0.05 was considered to indicate statistical significance.

## Electronic supplementary material

Supplementary files
