# Supplementary material for: Athyrium multidentatum (Doll.) Ching extract induce apoptosis via mitochondrial dysfunction and oxidative stress in HepG2 cells
Source: Sci Rep. 2017 May 23;7:2275. doi: 10.1038/s41598-017-02573-8 (PMC5442098; doi:10.1038/s41598-017-02573-8)

Supplementary files

Athyrium multidentatum (Doll.) Ching extract induce apoptosis via mitochondrial dysfunction and oxidative stress in HepG2 cells

*Guoyuan Qi, Zhigang Liu, Rong Fan, Ziru Yin, Yashi Mi, Bo Ren, Xuebo Liu**

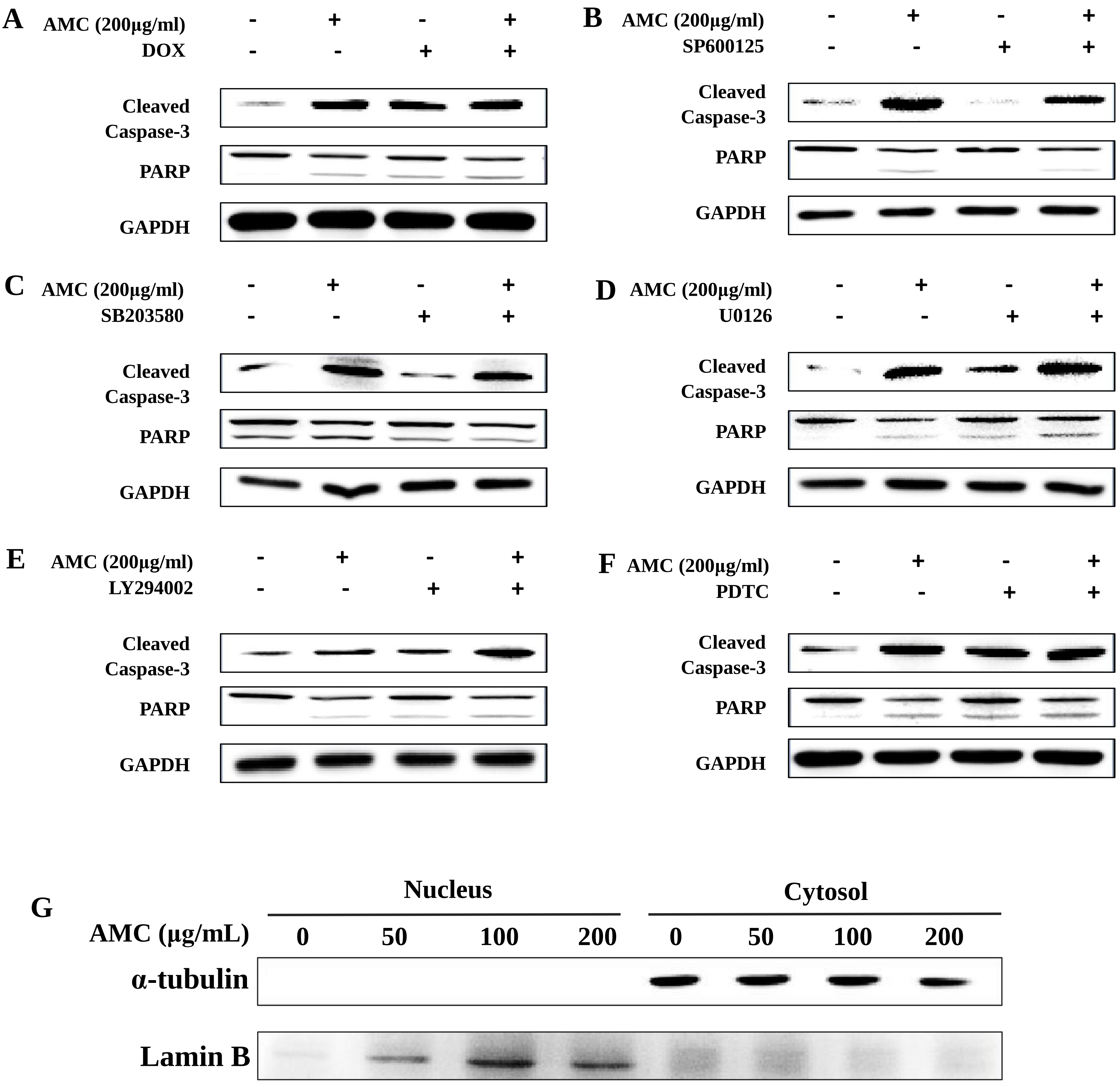

Supplement: Supplementary file 1 — Supplementary files [file 41598_2017_2573_MOESM1_ESM.pdf]
